# New Books

**Published:** 2007-01

Air Pollution XIV

J.W.S. Longhurst, ed.

Billerica, MA:WITpress, 2006. 824 pp. ISBN: 1-84564-165-5, $423

Chemicals as Intentional and Accidental Global Environmental Threats

Lubomir Simeonov, Elisabeta Chirila, eds.

New York:Springer, 2006. 514 pp. ISBN: 1-4020-5096-8, $219

Chernobyl—What Have We Learned?

Yasuo Onishi, Oleg V. Voitsekhovich, Mark J. Zheleznyak, eds.

New York:Springer, 2006. 289 pp. ISBN: 1-4020-5348-7, $129

Comparative Environmental Politics

Jerry McBeath, Jonathan Rosenberg

New York:Springer, 2006. 193 pp. ISBN: 1-4020-4762-2, $125

Energy and Culture: Perspectives on the Power to Work

Brendan Dooley

Burlington, VT:Ashgate, 2006. 264 pp. ISBN: 0-7546-4514-2, $114.95

Fluoride in Drinking-water

J. Fawell, K. Bailey, J. Chilton, E. Dahl, et al.

Geneva:WHO Press, 2006. 144 pp. ISBN: 9241563192, $63

Fluorine and the Environment

Alain Tressaud, ed.

Burlington, MA:Elsevier, 2006. 296 pp. ISBN: 0-444-52672-2, $165

Generation Extra Large: Rescuing Our Children from the Epidemic of Obesity

Chris Woolston, Lisa Tartamella, Elaine Herscher

New York:Perseus Books Group, 2006. 255 pp. ISBN: 0-465-08391-9, $14.95

Governance of Biodiversity: Conservation in China and Taiwan

Gerald A. McBeath, Tse-Kang Leng

Northampton, MA: Edward Elgar Publishing, 2006. 256 pp. ISBN: 1-84376-810-0, $99

Growing Smarter: Achieving Livable Communities, Environmental Justice, and Regional Equity

Robert D. Bullard, ed.

Cambridge, MA:MIT Press, 2006. 408 pp. ISBN: 0-262-02610-4, $67

Health of the People: The African Regional Health Report

World Health Organization

Geneva:WHO Press, 2006. 196 pp. ISBN: 929-023103-3, $27

Health Risks from Dioxin and Related Compounds: Evaluation of the EPA Reassessment

Committee on EPA’s Exposure and Human Health Reassessment of TCDD and Related Compounds, National Research Council

Washington, DC:National Academies Press, 2006. 268 pp. ISBN: 0-309-10258-8, $54

Politics of Oil: A Survey

Bulent Gokay, ed.

Oxford, UK:Routledge, 2006. 260 pp. ISBN: 1-8574-3340-8, $230

Protecting Ground Water for Health

World Health Organization

Geneva:WHO Press, 2006. 697 pp. ISBN: 924-154668-9, $144

Reality Check: The Nature and Performance of Voluntary Environmental Programs in the United States, Europe, and Japan

Richard D. Morgenstein, William A. Pizer, eds.

Washington, DC:RFF Press, 2006. 200 pp. ISBN: 1-933115-36-X, $80

Solar Revolution: The Economic Transformation of the Global Energy Industry

Travis Bradford

Cambridge, MA:MIT Press, 2006. 248 pp. ISBN: 0-262-02604-X, $24.95

Sustainable Nuclear Power

Galem Suppes, Truman Storvick

Burlington, MA:Elsevier, 2006. 416 pp. ISBN: 0-12-370602-5, $59.95

Sustainability or Collapse?

Robert Costanza, Lisa J. Graumlich, Will Steffen, eds.

Cambridge, MA:MIT Press, 2006. 520 pp. ISBN: 0-262-03366-6, $38

Why Care for Nature?

Dirk Willem Postma

New York:Springer, 2006. 221 pp. ISBN: 1-4020-5002-X, $129

